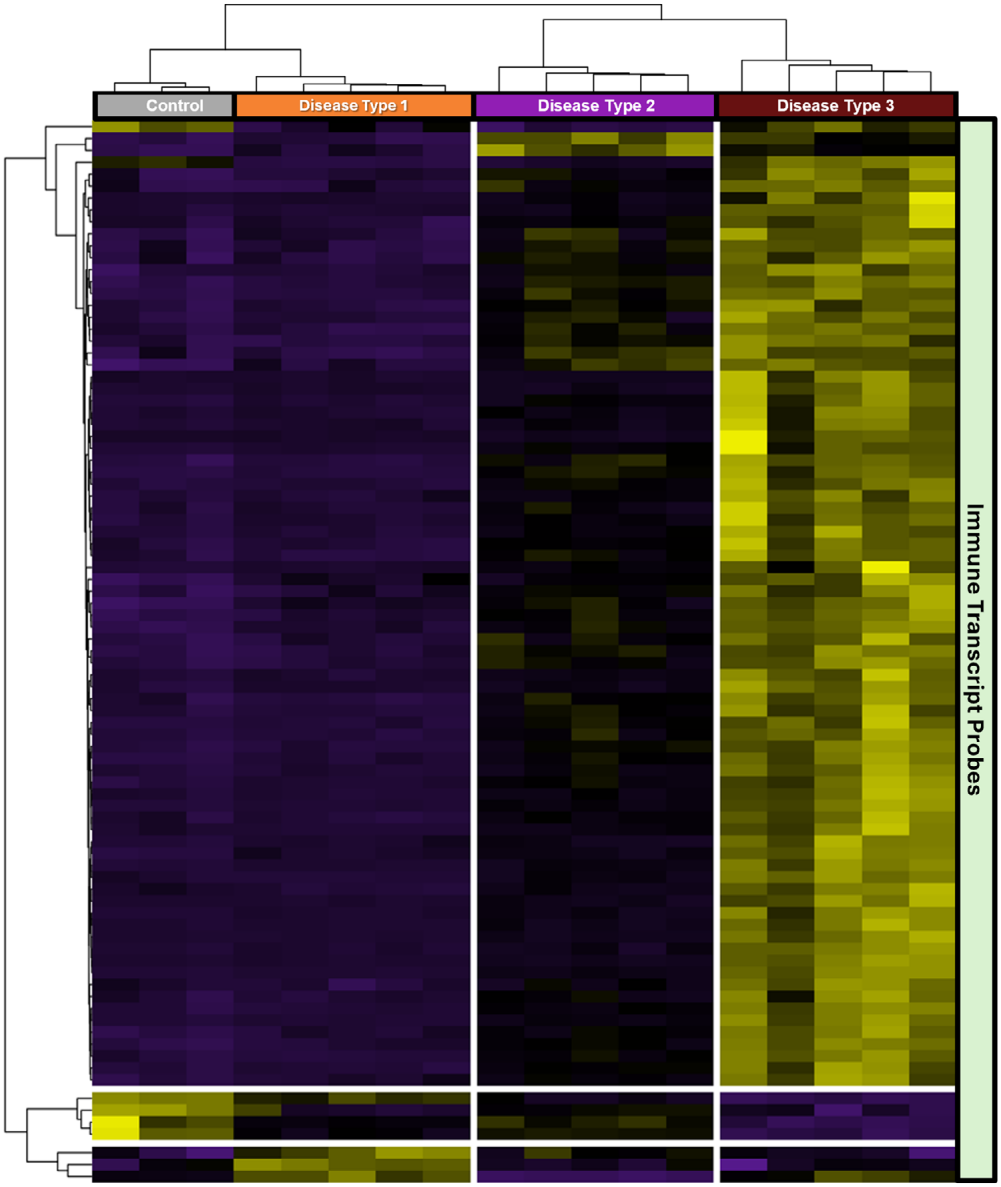# Spatially resolved transcriptomics/proteomics identifies increased inflammatory response in Alzheimer’s disease patients with Cerebral Amyloid Angiopathy

**DOI:** 10.1002/alz70862_110240

**Published:** 2025-12-23

**Authors:** Juan E Maldonado Weng, Harun N Noristani, Carmen E Schuldt, Pascale N Lacor

**Affiliations:** ^1^ Imaging Research and Development, Lilly Research Laboratories, Indianapolis, IN USA

## Abstract

**Background:**

Cerebral amyloid angiopathy (CAA) is a major co‐pathology exhibited by over 80% of Alzheimer’s disease (AD) patients. This neuropathological hallmark is defined by an accumulation of amyloid deposits in cerebral cortical and leptomeningeal vessels. Although pathogenesis is poorly understood, CAA leads to perivascular leakage, microaneurysms and an increased risk of cerebral hemorrhages, along with an elevated neuroinflammatory response. To identify CAA‐specific inflammatory pathways, we conducted ‐omics studies using the GeoMx® digital spatial profiler (DSP) in pathology‐enriched microenvironments such as amyloid deposition (plaques, parenchymal and leptomeningeal CAA) in postmortem human brain regions from AD patients with and without CAA.

**Method:**

Using the comprehensive whole transcriptome atlas (WTA) targeting 18,000 transcripts, we analyzed gene expression of pathologically relevant pathways in precise microenvironments across two preservation modalities (formalin‐fixed paraffin‐embedded vs fresh frozen). Additionally, we conducted GeoMx spatial profiling with neuro‐ and immune‐specific protein panels, on regions of interest (ROI) designed with the Visiopharm image analysis platform, affording greater cell‐type specific resolution.

**Result:**

Across both preservations, 7090 genes were detected in at least 10% of ROIs; however, over +2000 genes were uniquely detected in Frozen, while only 740 unique genes in FFPE tissue. Across Frozen tissue, the gene expression profile of AD+CAA estimated fewer neuronal and an increase in pericyte and astrocyte markers, compared to AD and control. Further, enrichment analysis demonstrated that, within amyloid plaque enriched microenvironments, AD+CAA expression profile was associated with vasculature development, T cell activation and inflammatory response, while AD was associated with synaptic and mitochondrial function. GeoMx proteomic assessment of 120+ proteins confirmed the upregulation of activated microglia and reactive astrocytes within amyloid enriched microenvironments with additional differentiation in parenchymal CAA, as evident by an enrichment of glial fibrillary acidic protein, vimentin and complement C4B. Additionally, perivascular immune cells surrounding leptomeningeal vessels with CAA exhibited the characteristics of cytotoxic T cells.

**Conclusion:**

Based on our findings, reactive astrocytic and macrophagic markers are potential valuable biomarkers of CAA in AD. Therefore, this study provides valuable insights into the inflammatory pathways specific to CAA, elucidating our understanding of this AD co‐pathology.